# Frequency and Predictors of Pacifier Use in the Low Socioeconomic Group of Karachi, Pakistan: A Cross-Sectional Study

**DOI:** 10.7759/cureus.12324

**Published:** 2020-12-27

**Authors:** Ramsha Riaz, Madiha Ahmed, Mariam Baloch, Syed Asad Hasan Rizvi, Naureen Shahid, Fatima Qadir Siddiqui, Muhammad S Haq, Sania Saleem

**Affiliations:** 1 Internal Medicine, Dr Ruth KM Pfau Civil Hospital Karachi, Dow University of Health Sciences, Karachi, PAK; 2 Medicine, Dr Ruth KM Pfau Civil Hospital Karachi, Dow University of Health Sciences, Karachi, PAK; 3 Internal Medicine, Dow University of Health Sciences, Karachi, PAK; 4 Internal Hospital, Dr Ruth KM Pfau Civil Hospital Karachi, Dow University of Health Sciences, Karachi, PAK; 5 Internal Medicine, Dow Medical College, Dow University of Health Sciences, Karachi, PAK; 6 Research and Development, California Institute of Behavioral Neurosciences & Psychology, Fairfield, USA; 7 Internal Medicine, King Edward Medical University, Mayo Hospital, Lahore, PAK

**Keywords:** pacifiers, breast feeding, paediatrics, feeding infants and young children, pakistan, developing countries

## Abstract

Background

Pacifier use has been popular for ages. They are prevalent all over the world because of their various perceived benefits. On the other hand, there is a common belief that they do carry health risks as well. Due to these contradicting belief systems, the frequency of their use, and the factors that determine them, need to be thoroughly evaluated. Since Pakistan is a developing country with a low literacy rate, it can be implied that a vast majority of the country’s population may lack awareness regarding the advantages and disadvantages of pacifier use, making them incapable of weighing associated risks versus benefits. The data evaluating these factors in this region are however scarce in the published literature. We, therefore, aimed to highlight the frequency and predictors of pacifier use in the low socioeconomic group of Karachi, Pakistan.

Materials and methods

A descriptive, cross-sectional study was conducted on a sample of 300 mothers visiting a tertiary care hospital in Karachi, Pakistan. We included mothers who had at least one child under the age of two years, and whose child did not have any oro-nasal anomaly that could prevent them from sucking a pacifier. Data were collected using pre-tested questionnaires and analyzed using the Statistical Package for Social Sciences (SPSS version 23.0, IBM Corp., Armonk, NY, US). Frequencies were calculated and presented in the form of tables. The chi-square test was used to determine the significance of all categorical variables. A P-value of <0.05 was considered to be statistically significant.

Results

Almost half of our respondents (49%) gave pacifiers to their children. A significant number (59%) of these mothers were uneducated. Almost all (97%) of the users had annual household income less than 15,000 Pakistani rupees (PKR); 34% were primiparous and more than two-thirds (71%) had a normal vaginal delivery. Out of all the factors, maternal age less than 20, annual household income less than 15,000 PKR, and primiparity were significantly associated with pacifier use in mothers (P<0.05). Only a half of the users (51%) cleaned the pacifiers by boiling; one-fourth (25%) washed it with water only; while 18% washed it with soap and water. The majority (84%) of the mothers used the pacifier to soothe the baby when upset. Among mothers who did not use a pacifier, about a third (30%) did not do so as they believed it’s a bad practice. About one-fourth (27%) believed it was unhygienic.

Conclusions

Our study highlights the gap in the awareness of mothers regarding pacifier use. Using this data, we can target to disseminate specific information to this population to integrate safe and healthy child care habits in society.

## Introduction

The topic of pacifier use has long remained a controversial subject in the field of pediatrics. Pacifiers, alternatively referred to as soothers, dummies, and artificial teats, are tiny devices made up of a silicone or latex nipple attached to a plastic shield and a handle [1]. They are commonly used across the globe for their various perceived benefits, while avoided in other parts for their risks and negative associations. The use of pacifiers is widespread in various communities as a means of soothing and relaxing the baby, relieving pain by functioning as an analgesic, and satisfying the baby’s innate non-nutritive suckling (NNS) response [2]. In addition, a meta-analysis has revealed the beneficial effect of pacifiers in shortening the duration of hospital stay in preterm infants [3]. Pacifiers have also been implicated to decrease the risk of sudden infant death syndrome (SIDS) [4]. Although the exact mechanism by which they achieve this is not completely understood, it is suggested that the use of pacifiers may improve the patency of the airways during sleep, prevent the infant from rolling into a prone position, promote cardiovascular stability, and improve autonomic breathing control [5].

In contrast to their benefits, pacifiers also have detrimental associations that have limited their use. These include increasing the risk for the development of otitis media, possible disruption of breastfeeding, and the development of nipple confusion and teeth malocclusion in certain cases [6]. It has been suggested that pacifier use can cause otitis media through Eustachian tube dysfunction and the reflux of nasopharyngeal secretions into the middle ear [2,6]. In one study, it was demonstrated that pacifier use resulted in a reduced frequency of breastfeeding by 26% [7]. Cases of latex allergy have also been reported with the use of pacifiers [8]. Furthermore, they are also an important source of infection as they are home to many microorganisms [2].

Considering the two schools of thought regarding pacifier use, studies conducted to investigate their frequency of use among various populations are of particular significance. Within the developed part of the world, the frequency of pacifier use among first-time mothers in Australia is 79%, whereas, a study conducted in the United States of America revealed a prevalence of 72.3% among African American mothers [9,10]. In the subcontinent, cultural motherhood practices make their use prevalent, especially among the lower socioeconomic class, therefore increasing the likelihood of pacifier associated complications occurring in these children [11].

In Pakistan, 60 million people, accounting for up to 29.5% of the country’s entire population, live below the poverty line and earn less than 33,030 Pakistani rupees (PKR) per month [12]. In addition to this, Pakistan’s current literacy rate is 62.3%, which suggests that an estimated population of 60 million is uneducated in the country [13]. Due to these facts, it can be implied that the vast majority of the country's population may not be aware of the proper hygiene practices associated with pacifiers, including the advantages and disadvantages of pacifier use. Despite this, very few researches have been conducted regarding pacifier use in this population group, and even fewer studies that take into account all pacifier-related associations in Pakistan. Therefore, the primary objective of our study is to highlight the frequency of pacifier use among the said population. We also aim to identify the different reasons why people opt to use pacifiers and the predictors of their use among children. This will help provide physicians with a better framework of how to counsel their patients and how to reduce the potential complications associated with pacifier use.

## Materials and methods

We conducted a descriptive, cross-sectional study among mothers visiting the out-patient department (OPD) of three pediatric units in a tertiary care hospital in Karachi, Pakistan. The sample size was obtained using OpenEpi.com, with an anticipated frequency (p) of 83.6%, at a 95% confidence interval (CI) and 5% confidence limit [11,14]. A minimum sample size of 211 was obtained, however, to increase the accuracy of our results and account for improperly filled data forms, the sample size was increased to 300. The convenience sampling method was employed to select participants from the pediatric units, from a duration of August to September 2019. 

Included in our study were mothers who had at least one child under the age of two years, and whose child did not have cleft lip/cleft palate or any other oro-nasal deformity that could prevent them from sucking a pacifier. All mothers who met the inclusion criteria were recruited to fill out self-administered questionnaires. Written informed consent was obtained from all participants who agreed to be a part of the study, and the confidentiality and anonymity of the participants were maintained. 

To assess the frequency and predictors of pacifier use, a structured, self-reported questionnaire was designed after thorough research on the available literature. The questionnaire was divided into three main parts. The first part contained questions regarding the demographic information of the participants, such as the age of the mother, educational level, occupational status, residential area, socioeconomic status, and use of a pacifier for their child/children. It also assessed factors like the parity of the mother, her mode of delivery, the total number of deliveries, and her breastfeeding history, practices, and problems encountered if any. The second part of the questionnaire was for participants who used pacifiers. It assessed the reasons mothers started the use of pacifiers, as well as the details regarding the hygiene practices related to pacifier use, such as the method of cleaning the pacifier, frequency of cleaning, reason for cleaning pacifier, sharing of pacifier among other children, storage method of the pacifier, and need for replacement of pacifier. The last part of the questionnaire was for participants who did not use pacifiers, and it mainly explored the reasons for not using pacifiers. 

A pilot study was conducted on 15 participants before the start of the study to assess the questionnaire for any ambiguities and to enhance the clarity and validity of the final questionnaire. The expert opinion of a senior pediatrician was also sought to finalize the questionnaire. The questions were translated into the National language of Pakistan, Urdu, to ease understanding and increase the response rate of the participants. The questionnaire was then retranslated into the English language to document the accurate response. All data collected were entered into the Statistical Package for Social Sciences (SPSS version 23.0, IBM Corp., Armonk, NY, US) and presented in the form of frequencies and percentages using Microsoft Word 2016. The chi-squared test was employed to assess for significance among all categorical variables. A P-value of <0.05 was considered statistically significant.

## Results

Among a total of 300 mothers selected for this study, 146 (49%) admitted to using a pacifier for their children. Among those, 82 (56%) had a male child and 64 (44%) had a female child. A significant number (59%) of pacifier-using-mothers were uneducated and most (89%) were stay-at-home. Almost all (97%) of the users had annual household income less than 15,000 PKR; 50 (34%) were primiparous and more than two-thirds (71%) had a normal vaginal delivery. Out of all, maternal age less than 20, annual household income less than 15,000 PKR, and primiparity were the demographics significantly associated with pacifier use in mothers (P<0.05). Table 1 demonstrates the sociodemographic characteristics of participants based on pacifier use.

**Table 1:** Sociodemographic Characteristics of Participants Based on Pacifier Use Note: P-value calculated using chi-square; P<0.05 considered statistically significant. EBF: exclusive breastfeeding; NVD: normal vaginal delivery

Variable	Pacifier User	Non-User	P-Value
Total participants	146 (49%)	154 (51%)	
Child's Gender			>0.05
Male	82 (56%)	96 (62%)	
Female	64 (44%)	58 (38%)	
Mother's Age at Delivery (Years)			0.03
<20	26 (18%)	10 (6%)	
>20	120 (82%)	144 (94%)	
Mother's Educational Status			>0.05
Not educated	86 (59%)	52 (34%)	
Primary (Grade 1-5)	32 (22%)	18 (12%)	
Secondary (Grade 6-8)	14 (10%)	2 (1%)	
Matriculation (Grade 9-10)	8 (5%)	14 (9%)	
Intermediate (Grade 11-12)	2 (1%)	10 (6%)	
University/Graduate	4 (3%)	0 (0%)	
Mother's Occupational Status			>0.05
Stay at home	130 (89%)	148 (96%)	
Part-time job	10 (7%)	2 (1%)	
Full-time job	6 (4%)	4 (3%)	
Residential Area			>0.05
Urban	126 (86%)	138 (90%)	
Rural	20 (14%)	16 (10%)	
Household Income			0.01
<15,000 PKR	142 (97%)	132 (86%)	
>15,000 PKR	4 (3%)	22 (14%)	
Primiparity			0.01
Yes	50 (34%)	26 (17%)	
No	96 (66%)	128 (83%)	
Mode of Delivery			>0.05
NVD	104 (71%)	114 (74%)	
Scheduled c-section	32 (22%)	26 (17%)	
Emergency c-section	10 (7%)	14 (9%)	
Total Number of Live Births			>0.05
	138 (95%)	130 (84%)	
>Five	8 (5%)	24 (16%)	
Type of Feed			>0.05
EBF	54 (37%)	64 (42%)	
Bottle/formula feed	54 (37%)	52 (34%)	
Both	38 (26%)	38 (25%)	

Of the 146 mothers who used the pacifier, only a half 74 (51%) cleaned the pacifiers by boiling; one-fourth 36 (25%) washed it with water only; while 26 (18%) washed it with soap and water. More than one-third of 62 (42%) mothers cleaned the pacifier only once daily; while about a third 44 (30%) cleaned the pacifier two to three times only. Only about 15% (22) of mothers cleaned it before every use. Table 2 shows the pacifier-related hygiene practices among the users.

**Table 2:** Pacifier-Related Hygiene Practices Among Users

	Number of Users (Percentage) (N=146)
Method of Cleaning Pacifier	
Don’t clean it at all	8 (5%)
Wash it with water only	36 (25%)
Wash it with soap and water	26 (18%)
Wash it using soap and sponge/scrub/brush	10 (7%)
By boiling	74 (51%)
Frequency of Cleaning/Day	
Never	8 (5%)
Once	62 (42%)
Often (2-3 times)	44 (30%)
Frequently (>3 times)	18 (12%)
Very frequently (before every use)	22 (15%)
Reason for Cleaning Pacifier	
It looks dirty	52 (36%)
To maintain hygiene	86 (59%)
The child may fall ill	8 (5%)
To make it last longer and be in good shape	8 (5%)
Sharing of the Same Pacifier Among Other Children	
Yes	14 (4%)
No	140 (96%)
Storage Method of Pacifier	
Leave it lying open	56 (38%)
In a box	62 (42%)
In a cloth	10 (7%)
Under sunlight	24 (16%)
Other	2 (1%)
Need for Replacement of Pacifier	
When it’s damaged/broken	48 (33%)
When it’s misplaced/lost	16 (11%)
After few months	12 (8%)
When my child demands a new one	6 (4%)
Others	72 (49%)

No association of pacifier use was found with breastfeeding, the timing of first feed after delivery, or any issues related to breastfeeding. Table 3 further elaborates on these results.

**Table 3:** Association of Breastfeeding With Pacifier Use Note: P-value calculated using chi-square; P<0.05 considered statistically significant.

	Pacifier User (N=146)	Non-User (N=154)	P-Value
Breastfeeding	92 (63%)	102 (66%)	>0.05
Exclusive	54 (37%)	64 (42%)	
Non-exclusive	38 (26%)	38 (12%)	
First Feed After Delivery			>0.05
Within 2 hours	54 (37%)	52 (34%)	
Between 2-24 hours	30 (21%)	58 (38%)	
>24 hours	58 (40%)	38 (25%)	
Other	4 (3%)	6 (4%)	
Issues During Breastfeeding			>0.05
Problems in attachment	15 (16%)	12 (12%)	
Improper sucking	14 (15%)	10 (10%)	
Problems in continuous feeding	11 (12%)	10 (10%)	

**Figure 1:**
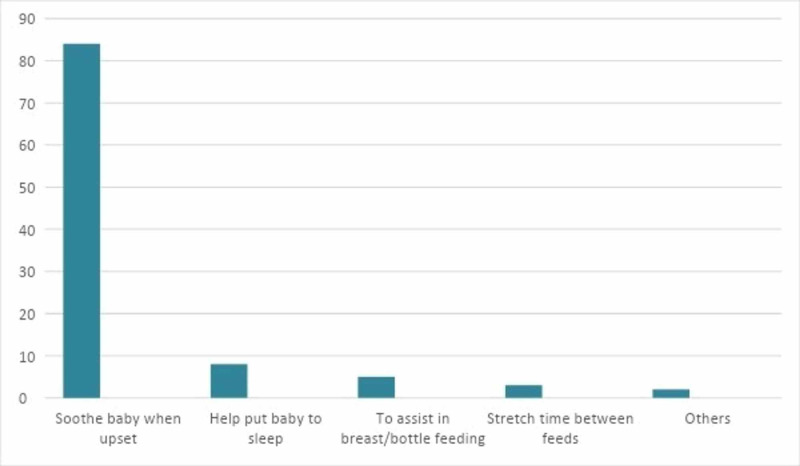
Reasons for Using Pacifiers (%)

The majority (84%) of the mothers used the pacifier to soothe the baby when upset. Not many used it to help put the baby to sleep, to assist in feeding, or to stretch the time between feeds. Figure 1 reveals the reasons for using a pacifier in the form of a bar chart.

**Figure 2:**
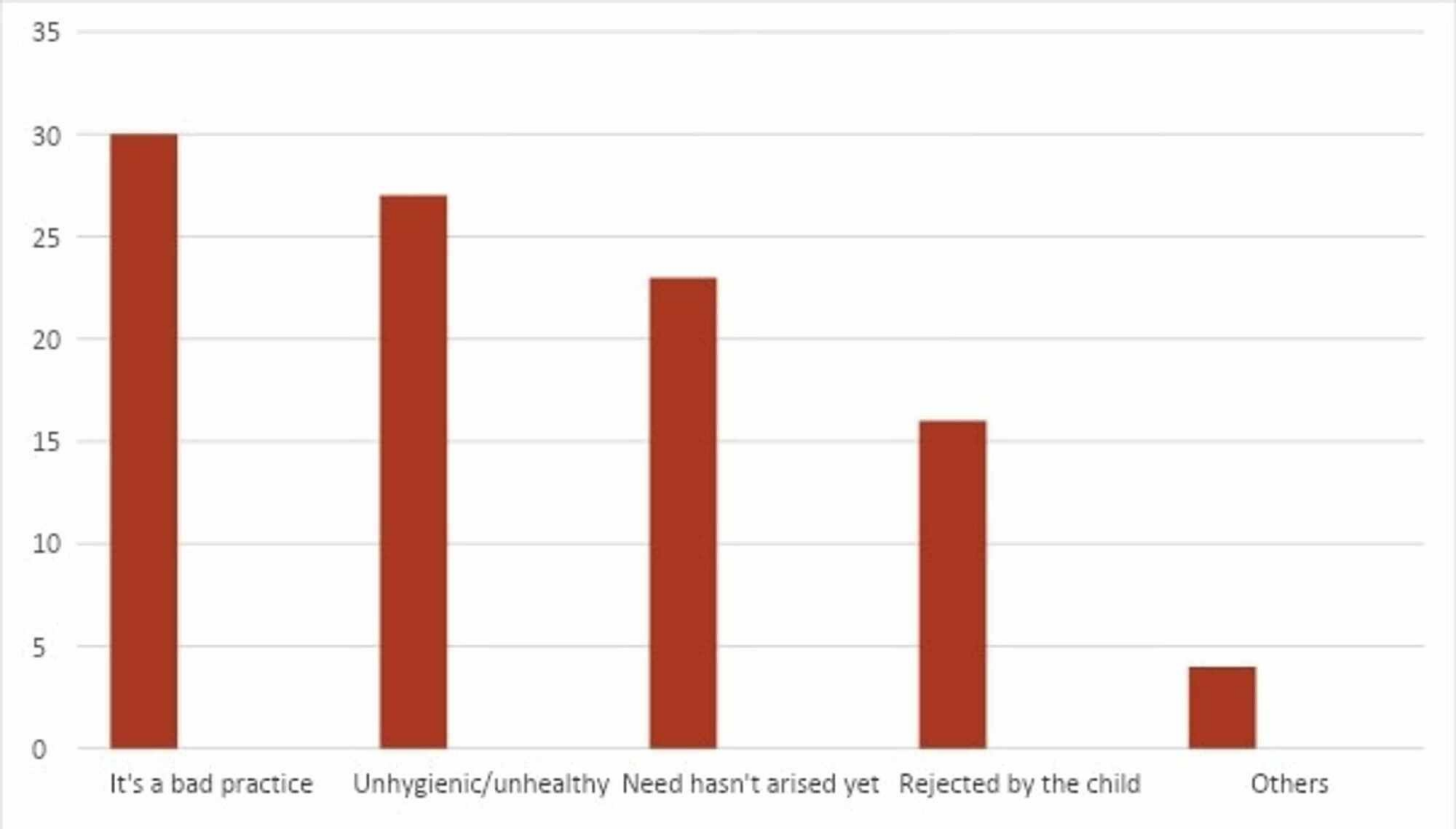
Reasons for Not Using Pacifiers (%)

Among mothers who did not use a pacifier, about a third (30%) did not do so as they believed it’s a bad practice. About one-fourth (27%) didn’t use a pacifier as they believed it was unhygienic. While the rest did not use it because either the need had not arrived yet or the child had rejected it. Figure 2 reveals the reasons for not using a pacifier.

## Discussion

Breastfeeding is endorsed universally owing to its health benefits and predominant role in reaching the global goals on nutrition, environmental sustainability, and economic growth [15]. As a point of fact, analysis reveals the additional US $300 billion to the world economy in a year on increasing the rates of breastfeeding [15]. Exclusive breastfeeding is recommended up to six months postpartum with initiatives in place to protect, promote, and support the practice [15]. Pacifier use is a controversial but modifiable risk factor that has been generally correlated to exclusive breastfeeding discontinuation [10,16-18]. Unfortunately, meta-analyses and systematic reviews evaluating the relationship between pacifier use and EBF duration have shown contradictory results [17,19]. Indeed, the systematic review by Jaafar et al. show “moderate quality” evidence of pacifier use is not significantly associated with the proportion of infants exclusively breastfed at three months (risk ratio {RR} 1.01; 95% CI 0.96 to 1.07, two studies, 1228 infants); and at four months of age (RR 1.01; 95% CI 0.94 to 1.09, one study, 970 infants) [19].

In addition to this, in a revised Baby-friendly Hospital Initiative (BFHI) Implementation guide of 2018, the World Health Organization has shifted its stance to discourage the use of pacifiers and artificial teats to endorse the statement, “counsel mothers on the use and risks of feeding bottles, teats, and pacifiers” [15]. This sparked confusion and debate about pacifiers’ use with countries laying down varying policies based on their local data [16]. On this account, our study evaluating the prevalence and determinants of pacifiers’ use in Pakistan is notable, adding useful evidence to the available literature.

In relation to the investigated event, almost half of our respondents (49%) gave pacifiers to their children. This is comparable to a study in Shiraz, Iran where pacifiers were given to 56.4% of the children [18]. The cognate prevalence can be attributed to the study site as both countries are neighbors in South Asia with fairly similar social and cultural norms. The overall prevalence of pacifier use in an investigation in Spain was 72.46%, whereas Nigeria, one of the poorest countries in the world, had a much lower usage of 12.6% attributed to unattainability as a result of cost [20,21]. In Australia, 79% of the first-time mothers had introduced pacifiers to their children mostly on the advice of their mother or mother-in-law, friends, other family members, husband or partner, and to a lesser extent by medical and other health professionals [9]. In the United States, a study conducted on African American women revealed that 72.3% of mothers gave pacifiers to their children [10]. In both the studies, the reasons for the use were quite similar to our survey, where the prime reason was to soothe the infant, followed by helping the baby to sleep, help in breastfeeding, and in some cases stretching the time between feeds [9,10]. The African American mothers thought that pacifiers made caring for the baby easier, especially at night when the mother wanted to sleep [10]. This is especially significant for single, working mothers without spousal support or working mothers in general who had work in the morning. A similar trend was observed in Australian mothers where 60% of them used a pacifier for settling the baby at night and helping him/her to sleep [9]. However, both crying and sleeping are frequent behaviors in babies, and thus can cause extensive or prolonged pacifier use. In fact, this is the reason for widespread and early use most days; though, it should be noted and subsequently taught to mothers that there are natural ways of soothing including carrying, rocking, and breastfeeding. These traditional attachment methods are not only more effective but help promote mother-baby bonding and consequent breastfeeding success [9].

The lesser popular reasons for using pacifiers in our survey were to aid in bottle or breastfeeding (5%) and to stretch the time between feeds (3%). Similar opinions were also recorded by the Australian mothers, where 6.8% used it for help in taking the baby off the breast, 0.9% used it for weaning the baby from breast to bottle, and 13% used it for stretching the time between feeds [9]. Feeding the infants on demand, especially the first few weeks of life, plays an important role in establishing the breast milk supply. Using a pacifier in this crucial period can interfere with the process potentially leading to shorter breastfeeding duration [9]. Prolonging the gaps between breastfeeding through pacifier reflects upon the mother’s desire of reprieve from the baby, and is consistent with prior literature where mothers practiced it to avoid feeling restricted with the baby or to leave the baby in someone else’s care [9,22]. This unveils that pacifier use is not solely based on infants’ needs. Dadalto and Rosa revealed that 37.1% of mothers used pacifiers because it helped them stay calm by making the infant stop crying [22]. For some African American mothers, it was aesthetically appealing to have the baby on a pacifier [10].

Regarding Pakistan, the socio-cultural setup is a possible underlying cause. In the traditional patriarchal setup, women are the homemakers with complete responsibility for household chores, ranging from cooking and cleaning to washing. Feeding the infant in these circumstances is sometimes not possible, bringing the pacifier into the equation. Public and mixed social gatherings where both genders are present is another pitfall. Exposing part of the body in front of other males is frowned upon, due to socio-religious beliefs. With no designated breastfeeding areas in most places, feeding either has to be accomplished by covering the child with a ‘dupatta’ (piece of cloth) or by opting for an alternative mode of feeding. In such cases, the infant is either bottle-fed, if it is hungry or given a pacifier, if irritable.

Women not using pacifiers in our study amounted up to 51% of the participants with most of them chalking it to be a bad practice (30%), or pacifiers being unhygienic and unhealthy (27%) for their child. This can be corroborated by the survey of Traebert et al. where dental malocclusions, including the presence of class II or III molar/canine relationship, posterior crossbite, and anterior open bite were significantly associated with pacifier use [23]. In another report, emergency room visits of an estimated 45,398 children under three years of age were linked to pacifiers (19.9%) [24]. Pacifiers were 3.04 times (95% CI: 1.97-4.72) more likely to injure a child under one year compared to two or three-year-old children [24]. Though, they still contributed to 18.1% of the injuries among two-year-old children [24]. Furthermore, pacifiers resulted in a higher likelihood of soft tissue injuries (OR: 1.86 {95% CI: 1.12-3.10}) or dental injuries (OR: 3.25 {95% CI: 1.75-6.04}) than bottles or sippy cups [24]. Mothers’ unwillingness to use pacifiers also stemmed from the additional cost of dental treatments as a result of malocclusions and change in the dental arch, maintenance of pacifier hygiene, and fear of bacterial contamination [22]. Contradictory to our study, the most common reason for pacifier non-use in African-American mothers was infant rejection [10].

Maternal age less than 20, household income less than 15,000 PKR, and primiparity constituted the socioeconomic factors significantly related to the use of pacifiers in our study (P<0.05). However, the outcome of studies by Bezerra et al. and Santos et al., respectively, contrasts with our findings with no significant link between the age of the mother and pacifier use [25,26]. Based on our understanding, a possible explanation lies in the fact that women younger than 20 may be inexperienced, newer to motherhood, and more easily impressionable by parenting suggestions passed around them. Therefore, they may feel overwhelmed by the new responsibilities of motherhood, or may not be able to adequately weigh the pros and cons of pacifier use. Pacifiers can relieve younger mothers for some time enabling them to perform all the necessary tasks and duties. With regards to the household income, 97% of respondents using a pacifier made a minimum monthly income of less than 15,000 PKR. Pineda et al. reported a similar trend with an income of less than US$ 25000 among the users (P=0.03) [27]. The conclusion drawn by Bezerra et al. did not follow suit with an insignificant P-value [25]. First-time mothers in our study were more likely to have their child suck on a pacifier, in contrast with the results presented by Bezerra et al. and Santos et al., where primiparity was not associated with pacifier use [25,26]. Other variables such as the mother’s educational status and her occupational status were not found to be significantly associated with pacifier use in our study (P>0.05). This contrasts with the pattern detected by Pineda et al., where pacifier use was less common in mothers who received a college education (P=0.03) [27]. On the other hand, the survey of Bezerra et al. revealed that lower maternal schooling had a negative association with the use of pacifiers (OR=0.47; P=0.045) [25]. 

The prime rationale behind many research studies focusing on pacifier use in the pediatric population is to investigate its impact on EBF. In lower-middle-income countries (LMIC), like Pakistan, where neonatal mortality is an issue, the promotion of EBF becomes extremely important. Even then, both breastfeeding and EBF rates do not meet the desired threshold. In our study, the overall practice of breastfeeding was 64.7%, with only 39.3% of the included mothers breastfeeding exclusively. Moreover, pacifier use among the children did not significantly relate to alteration in EBF rates. Conclusions drawn from other research studies portray a lack of consensus in this regard. Brazil has extensively focused on determinants of pacifier use in several studies, and the findings indicate pacifiers to be a barrier to EBF [16,17]. In fact, over a decade, an increase of 15.2% in exclusive breastfeeding came about in conjunction with a decrease of 17% in the prevalence of pacifiers among infants less than six months of age [16]. Approximately one-third of this improvement in EBF rates was, in effect, contributed to pacifier use [16]. Opposed to this, in a randomized clinical trial, early versus late introduction of pacifiers did not affect the prevalence of EBF at six months of age [28]. 

A faction of mothers in our study did not use pacifiers on account of hygiene concerns. For those that did use, cleaning practices of pacifiers were not satisfactory; only half of the mothers used boiling water, and a merely 18% used soap and water for cleaning, with a sizable portion of mothers using water only, and some not cleaning at all. Only 59% of mothers perceived the concept of cleaning to maintain hygiene and a considerable number of mothers left it lying around. The situation painted by these figures is disturbing, especially when it has been known that pacifiers can retain microorganisms [2]. An investigation has demonstrated that in vitro cleaning of pacifiers by alcohol rinse or microwave does not completely free pacifiers from microbes like *Candida albicans* and *Streptococcus mutans*, which are known to cause oral thrush and endocarditis in children respectively [29]. Not only that, possible contamination from viruses is a problem, especially in Pakistan where viruses like rotavirus, norovirus, and astrovirus can cause diarrhea and acute gastroenteritis as a result of an infection [30]. Keeping in mind that diarrhea resulting from rotavirus has caused 600,000 deaths of young children globally at one time and is still responsible for deaths of children in LMICs like Pakistan, it is crucial to disseminate information to mothers to sustain the hope of ever scaling down the high neonatal mortality [30].

Our study was not without its limitations. A small sample size meant that some of the observations cannot be generalized to a wider fraction of the population. Due to the limited amount of local literature and a self-designed questionnaire, some of the important determinants of pacifier use might have been omitted unintentionally. The study was conducted in an OPD which, in a typical tertiary care public hospital, means overcrowded waiting room. Despite our attempts to provide privacy, some mothers might have given socially acceptable answers to our questions on account of being heard and subsequently judged.

## Conclusions

Our study has played a pivotal role in bringing forth some eye-opening results, paving the path for future work in pediatrics with respect to pacifier use and current EBF trends, specifically in large cities like Karachi. There is limited literature available on pacifier use in Pakistan, therefore large scale studies are required to gather data for secondary analysis, that would lay the framework of local policies on ‘what’ to counsel mothers when explaining the ‘ten successful steps of breastfeeding’ to them. Primiparous mothers gather most of their information regarding child-care from their mothers, mothers-in-law, and other closely related female family members. As seen in our study, this could lead to suboptimal practices. Dissemination of authentic information by health-care professionals is needed to catalog the required change and integrate safe and healthy child-care habits in society.
